# Effect of Carbonate Chemistry Alteration on the Early Embryonic Development of the Pacific Oyster (*Crassostrea gigas*)

**DOI:** 10.1371/journal.pone.0023010

**Published:** 2011-08-10

**Authors:** Frédéric Gazeau, Jean-Pierre Gattuso, Mervyn Greaves, Henry Elderfield, Jan Peene, Carlo H. R. Heip, Jack J. Middelburg

**Affiliations:** 1 Centre National de la Recherche Scientifique-Institut National des Sciences de l'Univers, Laboratoire d'Océanographie de Villefranche, Villefranche-sur-Mer, France; 2 Université Pierre et Marie Curie-Paris 6, Observatoire Océanologique de Villefranche, Villefranche-sur-Mer, France; 3 Department of Earth Sciences, University of Cambridge, Cambridge, England; 4 Centre for Estuarine and Marine Ecology, Netherlands Institute of Ecology, Yerseke, The Netherlands; 5 Department of Marine Organic Biogeochemistry, Royal Netherlands Institute for Sea Research, Den Burg, The Netherlands; 6 Faculty of Geosciences, Utrecht University, Utrecht, The Netherlands; Heriot-Watt University, United Kingdom

## Abstract

Ocean acidification, due to anthropogenic CO_2_ absorption by the ocean, may have profound impacts on marine biota. Calcareous organisms are expected to be particularly sensitive due to the decreasing availability of carbonate ions driven by decreasing pH levels. Recently, some studies focused on the early life stages of mollusks that are supposedly more sensitive to environmental disturbances than adult stages. Although these studies have shown decreased growth rates and increased proportions of abnormal development under low pH conditions, they did not allow attribution to pH induced changes in physiology or changes due to a decrease in aragonite saturation state. This study aims to assess the impact of several carbonate-system perturbations on the growth of Pacific oyster (*Crassostrea gigas*) larvae during the first 3 days of development (until shelled D-veliger larvae). Seawater with five different chemistries was obtained by separately manipulating pH, total alkalinity and aragonite saturation state (calcium addition). Results showed that the developmental success and growth rates were not directly affected by changes in pH or aragonite saturation state but were highly correlated with the availability of carbonate ions. In contrast to previous studies, both developmental success into viable D-shaped larvae and growth rates were not significantly altered as long as carbonate ion concentrations were above aragonite saturation levels, but they strongly decreased below saturation levels. These results suggest that the mechanisms used by these organisms to regulate calcification rates are not efficient enough to compensate for the low availability of carbonate ions under corrosive conditions.

## Introduction

Due to the absorption of anthropogenic CO_2_ by the ocean, seawater pH has already declined by 0.1 unit compared with pre-industrial values [1] and is projected to decrease by another 0.35 unit by the end of the century [2]. This process, known as ocean acidification, will most likely have profound impacts on marine biota. Besides the direct effect of decreasing pH on the physiology and metabolism of marine organisms through a disruption of inter-cellular transport mechanisms [3], calcareous organisms are particularly sensitive due to the decreasing availability of carbonate ions (CO_3_^2−^) driven by increasing pCO_2_. The calcium carbonate saturation state (Ω) is defined as:
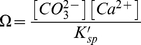
(1)where K′_sp_ is the stoichiometric solubility product, a function of temperature, salinity, pressure and the mineral phase considered (calcite, aragonite or high-magnesian calcite), and, as a consequence of ocean acidification, will significantly decrease in the coming decades. It must be stressed that carbonate saturation states depend not only on pH but also on total alkalinity levels. Total alkalinity measures the ability of a solution to neutralize acids to the equivalence point of carbonate or bicarbonate, acting as a natural buffer to the incorporation of anthropogenic CO_2_ in the ocean. As the addition (or removal) of CO_2_ to a solution does not change its alkalinity and since the dissolution of calcium carbonate minerals in the water column and in the sediments, as well as alkalinity inputs from continental rock weathering, are very slow processes, they are not expected to significantly buffer ocean acidification in the coming decades [4].

Several experimental studies have investigated the effect of a pCO_2_ increase on the growth of calcifying organisms [5]. Species that produce aragonite, less soluble than low-magnesian calcite in seawater, will be especially at risk. As amorphous calcium carbonate and aragonite have been identified as the main CaCO_3_ minerals in larval stages of benthic mollusks [6], there is a strong need to carefully assess the effects of ocean acidification and the associated alteration of the carbonate chemistry on their development. Small changes in the abundance and developmental success of these larval stages control the size and viability of the benthic populations [7] and therefore could induce significant changes in the functioning of coastal ecosystems. Indeed, shellfish are ecosystem engineers governing energy and nutrient flows in coastal ecosystems, provide habitats for many benthic organisms and form an important food source for birds [8], [9]. Moreover, global shellfish aquaculture production reached 13.1 million tons in 2008 (27% of the global aquaculture yield), corresponding to a commercial value of US$13.1 billion. The Pacific Oyster (*Crassostrea gigas*) was the most cultivated species in 2008 with a volume of 6.5 million tons or 9.5% of the total world aquaculture production (FISHSTAT Plus, Universal software for fishery statistical time series, Version 2.3, Food and Agriculture Organization of the United Nations, Fisheries Department, Data and Statistics Unit., 2000).

Several recent studies have focused on the effect of ocean acidification on the early development of molluscan species [10], [11], [12], [13], [14], [15], [16], [17], [18] and most of them have reported negative impacts of decreasing pH levels on the growth and development of these organisms. Kurihara et al. [12], [13], Parker et al. [15] and Gazeau et al. [11] have investigated the effects of decreasing pH on the early embryonic (from fertilization to the D-veliger stage) development of commercially important bivalve species. This developmental period is of the utmost importance since the onset of shell mineralization occurs during the trochophore larval stage and shells are fully mineralized when larvae reach the D-veliger stage, at the second or third day after fertilization [19]. Studies of Kurihara et al. [12], [13] on *Crassostrea gigas* and *Mytilus galloprovincialis* showed a strong decrease of developmental success into viable D-shaped larvae and growth rates with increased pCO_2_. However, a pH (on the National Bureau of Standards scale, hereafter referred to as pH_NBS_) of ∼7.4 was used (0.7 unit lower than control values), a value lower than that projected to occur at the end of this century. Moreover, due to low ambient total alkalinity levels, seawater was highly undersaturated with respect to aragonite in the low pH conditions in these two studies (Ω_a_ of 0.68 and 0.49, respectively). Parker et al. [15] studied the early embryonic development of the Sydney rock oyster *Saccostrea glomerata* at ambient (375 µatm), 600, 750 and 1000 µatm pCO_2_ levels. This experiment showed a general decrease in the percentage and size of D-veliger with increasing pCO_2_. However, manipulation of the carbonate chemistry was performed by addition of a strong acid (HCl) to reduce pH, a technique that is not recommended as acid addition also decreases total alkalinity, which is not anticipated to occur in the coming decades. Moreover, as values of total alkalinity and CaCO_3_ saturation state were not provided, comparisons with similar studies are not straightforward. Gazeau et al. [11] reported on the impacts of decreasing pH levels on the first 2 d development of blue mussel (*Mytilus edulis*) larvae. They showed that a decrease of pH_NBS_ to ∼7.8 (control: ∼8.1), associated with a supersaturation with respect to aragonite (Ω_a_∼1.4), had no effect on the percentage of embryos developing to viable D-veliger larvae. The effect on average final shell length was limited (∼−5±1%). Their results show that a decrease of pH_NBS_ to ∼7.6, associated with a slight undersaturation with respect to aragonite, had significant effects on the percentage of embryos that developed to D-veliger larvae normally and more pronounced effects on average final D-veliger shell lengths (∼−13±1%). However, this study did not allow the discrimination between the physiological effect of pH decrease, via a disruption of inter-cellular transport mechanisms, and the effect of the aragonite saturation state, on the larval development of this species.

The objectives of the present study are to investigate the effect of various carbonate chemistry alterations, performed by manipulating pH, total alkalinity and the saturation state with respect to calcium carbonate separately on the survival and growth of Pacific oyster (*Crassostrea gigas*) larvae during the first 3 days of their development.

## Materials and Methods

### Experimental set-up

**Figure 1:**
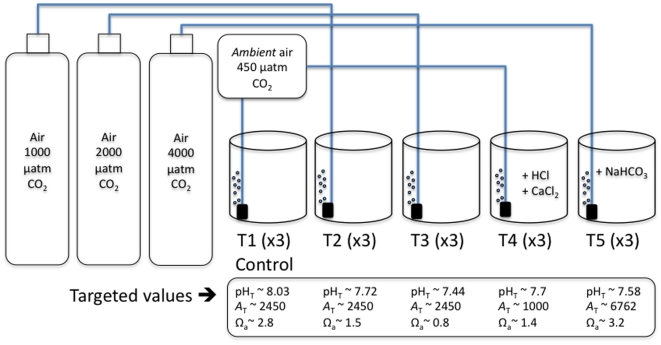
Experimental set-up. For each treatment (in triplicate; T = 18.9±0.1°C), the target A_T_ (total alkalinity in µmol kg^−1^), pH_T_ and Ω_a_ (saturation state of the seawater with respect to aragonite) are indicated. pH_T_ was controlled by bubbling ambient or high-CO_2_ air. A_T_ was decreased in T4 by HCl addition and increased in T5 by NaHCO_3_ addition. In T4, calcium concentrations have been increased above ambient levels by CaCl_2_ addition. See text for more details.

A batch of one million embryos of the Japanese oyster (*Crassostrea gigas*) was provided by the commercial hatchery *Roem van Yerseke* (Yerseke, The Netherlands) on 3 June 2009. This batch was transported within 15 min to a temperature-regulated room (19°C) at the *Netherlands Institute of Ecology* (Yerseke, The Netherlands) and evenly distributed into 15 beakers of 4.5 l (larval concentration of ∼15 ind. ml^−1^), containing filtered (0.2 µm) seawater from the Oosterschelde, the nearby tidal inlet. Five treatments were considered, each of them in triplicate (see Fig. 1 for the experimental setup). One treatment served as a control i.e. beakers were gently bubbled with external ambient air. Two beakers were bubbled with air at 1000 and 2000 µatm of CO_2_ (T2 and T3, respectively). The fourth treatment (T4) was bubbled with external ambient air, after total alkalinity (*A*_T_) was decreased to ∼1000 µmol kg^−1^ by adding 14 ml of HCl 0.1 N and 10.6 g of CaCl_2_-2H_2_O in order to reach saturation states with respect to aragonite and calcite of 1.4 and 2.2, respectively. The last group of beakers (T5) was bubbled with 4000 µatm CO_2_ and *A*_T_ was increased by adding 1.6 g of NaHCO_3_. Gas cylinders with certified CO_2_ concentrations (1000, 2000 and 4000 µatm) were supplied by Westfalen. Embryos were allowed to develop, without additional feeding, until larvae reached the shelled D-veliger stage, i.e. 72 h.

### Carbonate chemistry measurements

Seawater pH (on the total scale, hereafter referred to as pH_T_) and temperature were measured twice a day in the beakers. pH_T_ was measured using a pH meter (Metrohm, 826 pH mobile) with a glass electrode (Metrohm, electrode plus) calibrated on the total scale using Tris/HCl and 2-aminopyridine/HCl buffer solutions with a salinity of 35.0 [20]. Samples for salinity and *A*_T_ were taken at the start and at the end of the experiment. Samples for *A*_T_ were filtered on GF/F filters, poisoned with HgCl_2_ and stored in the dark pending measurement (within few days). Salinity was measured using a conductimeter (Radiometer CDM230). Triplicate potentiometric measurements of *A*_T_ were performed using a Metrohm titrator and a glass electrode (Metrohm, electrode plus). Measurements were carried out on 50 ml samples at 25°C and *A*_T_ was calculated using a Gran function. Titrations of *A*_T_ from standard seawater provided by A. G. Dickson (batch 82, n = 10) were on average within 0.46 µmol kg^−1^ of the nominal value. All parameters of the carbonate chemistry were determined from pH_T_, *A*_T_, temperature and salinity using the R package seacarb [21].

### Sampling and measurements

At the end of the incubation period, the beakers were emptied. Two liters were passed through a 30 µm sieve and concentrated in 50 ml that was fixed in a 5% neutralized-formalin seawater solution to determine developmental success (% of D-veliger larvae) and D-veliger shell length and area. The developmental success into viable D-shaped larvae was defined as the percentage of “normal” D-shape larvae following the criteria proposed by His et al. [22], after observation of at least 500 larvae per replicated culture. D-veliger larvae shell length (anterior to posterior dimension of the shell parallel to the hinge) and area were measured on 100 larvae per replicate based on pictures taken under a microscope (20×; 0.01 µm precision in length measurement), using the software Leica Qwin Pro version 2.4. At the end of the experimental period, 2 l of the cultures were filtered onto GF/F filters for subsequent analyses of calcium (Ca^2+^) concentrations. Larvae-free seawater was filtered onto GF/F filters (4×) and served as blanks for Ca^2+^ measurements. Ca^2+^ concentrations were determined by inductively coupled plasma emission spectrophotometry (ICP-OES) after multiple rinses with deionised water to remove seawater Ca^2+^. In addition to Ca^2+^ from the larvae, Ca^2+^ retained on the filters may come from residual sea salts, from the GF/F filters themselves and/or from the analytical blank of the procedure. To separate the contribution to total Ca^2+^ from these components, the filters were rinsed by soaking three times in 30 ml deionised water, increasing the duration each time, with approximately 10 sec allowed for the first rinse, 25 min for the second and an hour for the third. The water from each rinse was analyzed for Ca^2+^ and Na^+^ to determine the seawater contribution, then acidified to ∼0.1 M with nitric acid and reanalysed to determine the water insoluble component removed from the filters during rinsing. After the third rinse, remaining calcium was dissolved from the filters by immersing in 30 ml 0.1 M nitric acid for 30 minutes and the concentrations determined by ICP-OES. Ca^2+^ concentrations were then corrected for the concentration of Ca^2+^ observed on the blank filters following the same procedure and the total acid soluble Ca was calculated. As only one filter per treatment was analysed, the standard deviations associated with these measurements presented in the next section correspond to those for the blank filters. Calcium concentrations were normalized by the amount of eggs inoculated into the beakers at the start of the experiment, assuming that the distribution of the original batch has been performed homogeneously.

### Statistics

Since normality and homoscedasticity tests could not be used due to the small number of replicates (3), differences in percentage of viable D-shaped larvae, final shell lengths and areas as well as in the amount of calcium incorporated between the different treatments were tested by means of Kruskal-Wallis tests and post-hoc Dunn's multiple comparison tests (Graphpad Instat software). For all tests, differences were considered significant at p<0.05. In the following section, data are presented as means ± SD. In order to relate the different measured parameters to the carbonate chemistry parameters at which the organisms were exposed during the incubations, linear and non-linear regressions were performed and the significance of these relationships was tested using student's t-tests.

## Results and Discussion

**Figure 2:**
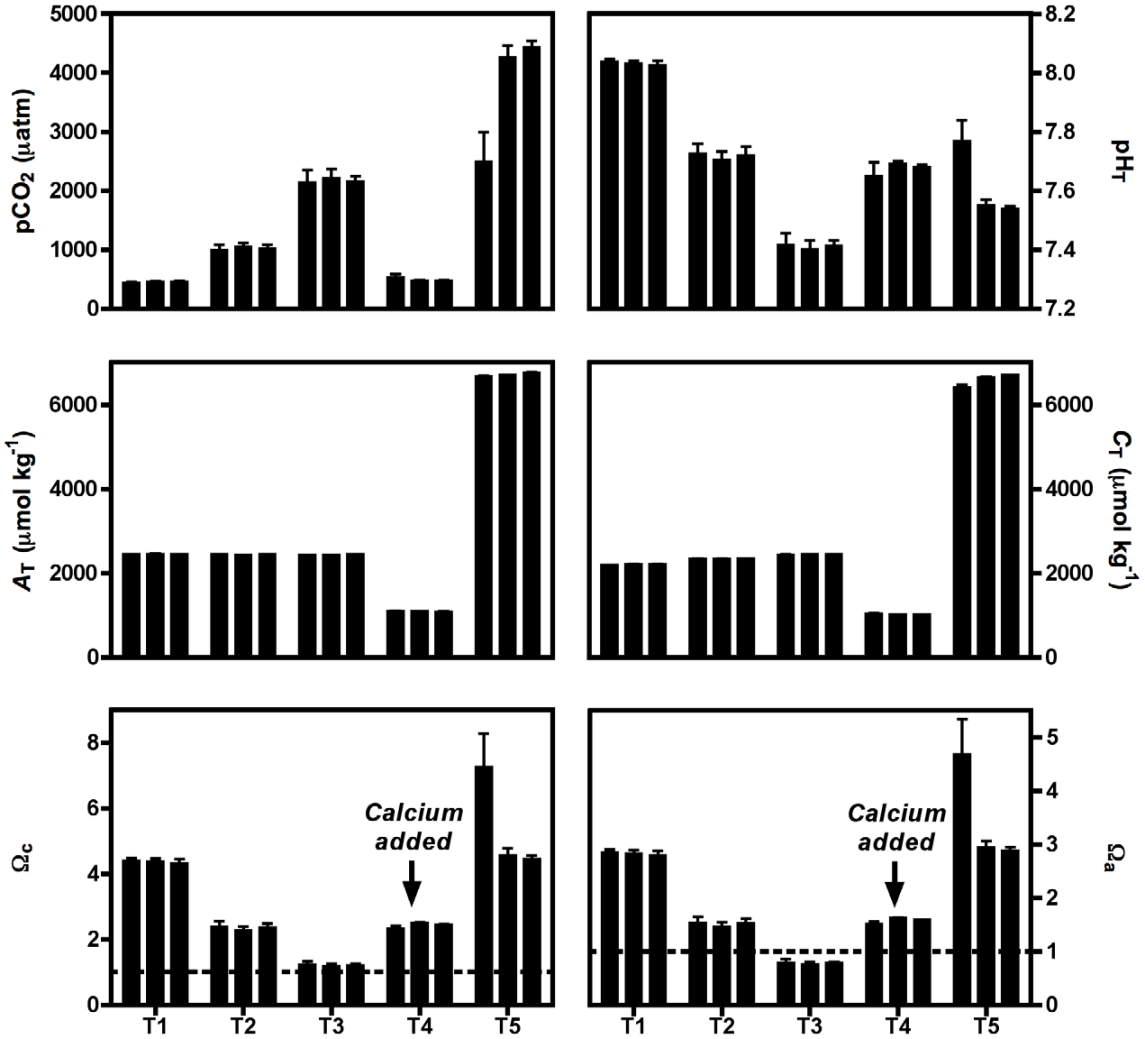
Carbonate chemistry conditions during the experimental period. pCO_2_: partial pressure of CO_2_, pH_T_: pH on the total scale, *A*_T_: total alkalinity, *C*_T_: dissolved inorganic carbon, Ω_a_ and Ω_c_: saturation state of the seawater with respect to, respectively, aragonite and calcite.

The environmental (temperature and salinity) and carbonate chemistry parameters are shown in Table 1 for each treatment. Parameters of the carbonate chemistry are also presented in Fig. 2 for each experimental beaker. Temperature was constant in the beakers at 18.9±0.1°C. Salinity was 34±0.1 in the first three treatments while it was slightly higher in T5 (34.3±0.0) due to the addition of NaHCO_3_ and more than 1 unit higher in T4 due to CaCl_2_ addition (35.4±0.1). pCO_2_ values were close to target values in most cases, except for one experimental beaker of T5 in which bubbling was not optimal and pCO_2_ was much lower than the expected value (2502 vs. 4000 µatm). pH_T_ varied from 8.03±0.01 in the control treatment (T1) to 7.41±0.03 in the beakers that were bubbled with 2000 µatm CO_2_ enriched air (T3). *A*_T_ was similar in the first 3 treatments while it has been successfully decreased to ∼1000 µmol kg^−1^ in T4 and increased to ∼6800 µmol kg^−1^ in T5. Total dissolved inorganic carbon (*C*_T_) concentrations were the lowest in T4 (1029±9 µmol kg^−1^) and the highest in T5 (6589±134 µmol kg^−1^) with variable levels within the beakers due to the non-optimal CO_2_ equilibration. Seawater was supersaturated with respect to calcite in all treatments with the lowest value for T3 (1.2±0.1). Undersaturation with respect to aragonite was observed in T3 (0.8±0.1) while the other 4 treatments showed Ω_arag_ values over 1, with a level of 1.6±0.0 in T4 due to addition of CaCl_2_.

**Table 1:** Environmental parameters and carbonate chemistry for the five different treatments during the course of the experiment (mean ± SD).

	T1 (control)	T2	T3	T4	T5
**Measured parameters**					
Temperature (°C)	18.9±0.1				
Salinity	34.0±0.1	34.1±0.1	34.1±0.1	35.4±0.1	34.3±0.0
pH_T_	8.03±0.01	7.72±0.03	7.41±0.03	7.67±0.03	7.62±0.12
*A*_T_ (µmol kg^−1^)	2452.7±6.6	2446.2±8.2	2443.1±3.1	1093.8±4.0	6726.6±37.6
**Computed parameters**					
pCO_2_ (µatm)	448.7±15.6	1019.9±79.9	2170.5±156.9	493.5±42.7	3730.4±946.5
*C*_T_ (µmol kg^−1^)	2207.0±10.1	2340.8±12.3	2443.4±10.6	1029.4±8.9	6589.3±133.9
[HCO_3_^−^]	2010.0±13.0	2209.4±15.1	2320.3±8.6	972.9±9.8	6238.0±161.9
[CO_3_^2−^]	181.9±4.2	97.3±6.3	50.4±3.3	40.0±2.4	226.5±60.3
Ω_a_	2.8±0.1	1.5±0.1	0.8±0.1	1.6±0.0*	3.5±0.9
Ω_c_	4.4±0.1	2.3±0.2	1.2±0.1	2.4±0.1*	5.4±1.4

The partial pressure of CO_2_ (pCO_2_), dissolved inorganic carbon concentration (*C*_T_) as well as the saturation state of seawater with respect to aragonite and calcite (Ω_a_ and Ω_c_ respectively) were computed from pH_T_ and total alkalinity (*A*_T_).

*: Ω_a_ and Ω_c_ were increased by addition of calcium (CaCl_2_-2H_2_O; ×2.6 *in situ* Ca^2+^ concentrations).

**Figure 3:**
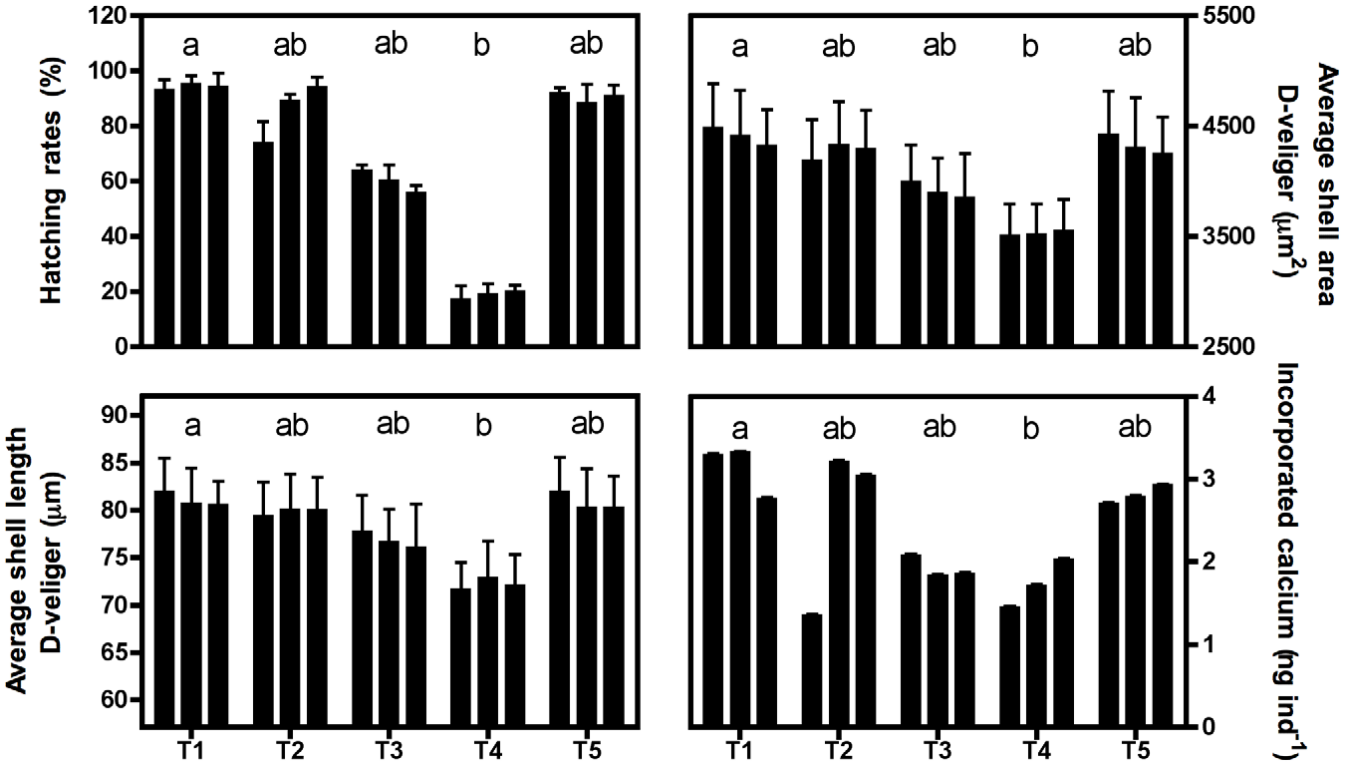
Larval developmental parameters at the end of the incubation period in the five treatments (72 h; T1 to T5). Proportion of embryos that developed to viable D-veliger (±SD; upper left plot), average shell area and length of D-veliger larvae (±SD; upper right and lower left plot, respectively) as well as the amount of calcium incorporated (±SE; lower right plot) are shown. Different letters on bars indicate significant differences in the median values (Kruskal-Wallis and post-hoc Dunn's multiple comparison tests, p<0.05).

**Figure 4:**
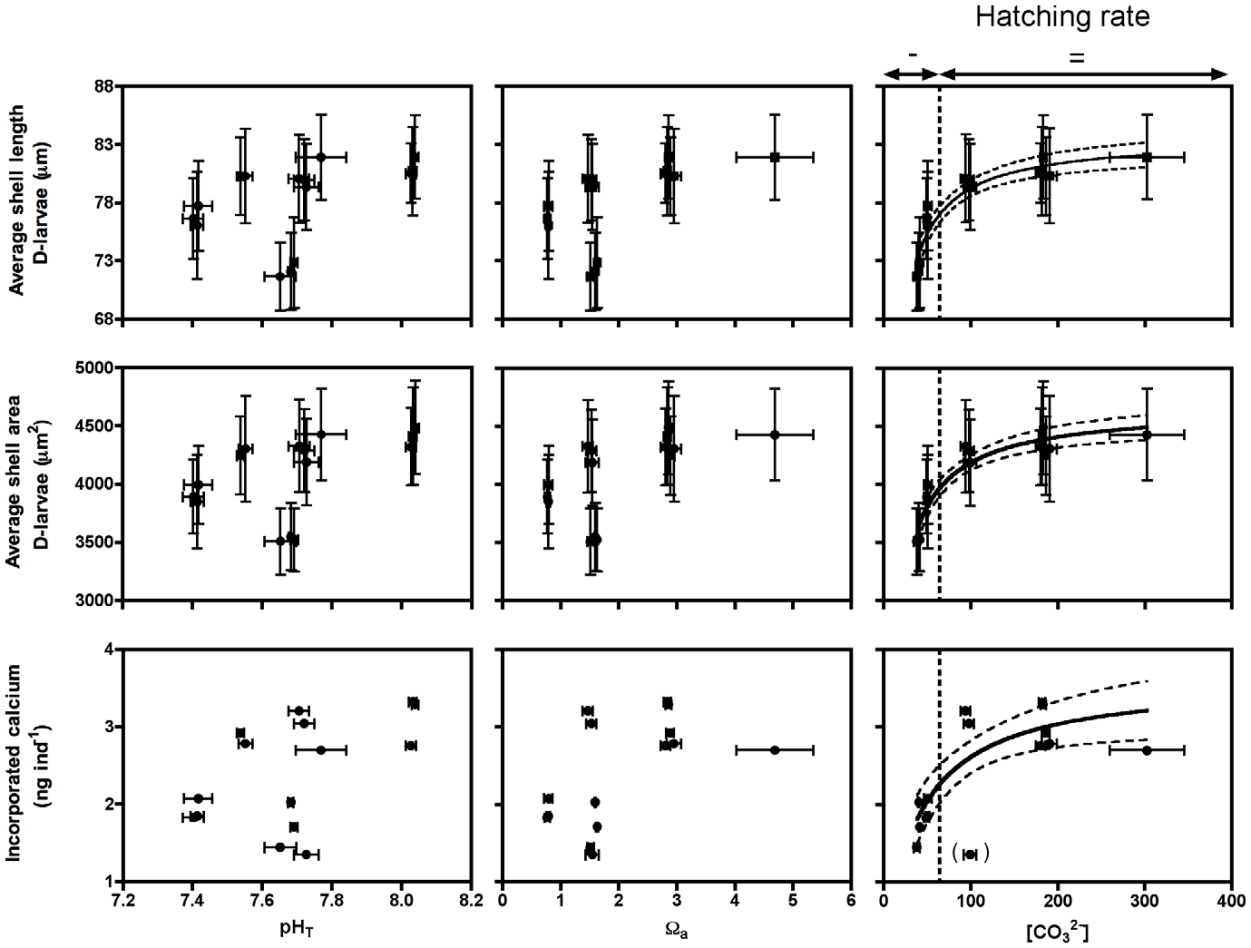
Relationships between larval developmental parameters and conditions of the carbonate chemistry in the five treatments. Relationships between the average (±SD) shell length and area of D-veliger larvae as well as the amount of calcium incorporated at the end of the 72 h incubation period, and the average (±SD) conditions of the carbonate chemistry in the five treatments are shown; pH_T_: pH on the total scale (left plots), Ω_a_: saturation state with respect to aragonite (middle plots) and [CO_3_^2−^]: carbonate ion concentration (right plots). On the right plots, the dotted lines refer to the carbonate ion concentration at the aragonite saturation level (see text for details).

Developmental success into viable D-shaped larvae, average D-veliger shell length and area as well as the amount of Ca^2+^ incorporated per egg inoculated, for the five different treatments, are shown in Fig. 3. Percentages of viable D-shaped larvae were 90% in the control treatment and not significantly different between T1, T2, T3 and T5, while significantly lower values were observed for T4 beakers (average of 19±1%). Final D-veliger shell length and area data showed the same pattern, with no significantly different values between T1, T2, T3 and T5 and significantly lower values in T4 as compared to the control treatment. Final D-veliger shell length and area were respectively 11±1% and 20±2% smaller in T4 as compared to control values. Significantly less Ca^2+^ was incorporated by the population in T4 than in the other treatments with a decrease of 45±14% with respect to control values. Final D-veliger shell length and area as well as the amount of Ca^2+^ incorporated are plotted against pH_T_ and Ω_a_ in Fig. 4. None of these parameters were correlated with pH_T_ or Ω_a_. Although seawater pH_T_ values in T4, T2 and T5 were similar at ∼7.65, the larvae were smaller in T4. Increasing the saturation state with respect to aragonite by adding Ca^2+^ (T4) did not positively affect the larval development since D-veliger shell length and area as well as the amount of Ca^2+^ incorporated were lower in T4 than in T2 which had similar Ω_a_ levels (i.e. ∼1.5). On one hand, no significant linear and/or non-linear relationships were found between all these parameters and pH or Ω_a_. On the other hand, these parameters were significantly correlated with the concentration of CO_3_^2−^ ions. Michaelis-Menten functions were used to fit the data (shell length: r^2^ = 0.90; shell area: r^2^ = 0.90; calcium incorporated: r^2^ = 0.74). Carbonate ion concentrations at the aragonite saturation level were estimated for each treatment (average of 64.4±0.2 µmol kg^−1^) and plotted as a dotted line on Fig. 4. Above the CO_3_^2−^ saturation level, the effects of decreasing CO_3_^2−^ concentrations on shell growth and Ca^2+^ incorporation as well as on the percentage of viable D-shaped larvae during these first 72 h of development were not significant (Fig. 4). Below the saturation level, decreasing CO_3_^2−^ concentrations resulted in smaller larvae, less Ca^2+^ incorporation in the shells and much lower developmental success (decrease in percentage of viable D-veliger larvae).

The present study is, to the best of our knowledge, one of the first to investigate the effects of carbonate chemistry modifications on the growth of a marine calcifier by separately assessing the effects of decreases in pH, carbonate ion availability and seawater saturation state with respect to calcium carbonate. Separating these potential factors is crucial as total alkalinity levels are not constant in the ocean and a similar decrease in pH does not lead to similar decline in calcium carbonate saturation state. A similar study has been performed recently by Jury et al. [23], based on manipulations of the seawater carbonate chemistry, to determine which parameter controls coral calcification. They showed that the calcification rate of *Madracis auretenra* is mainly governed by the bicarbonate ions concentration and not, as expected, by the aragonite saturation state. In contrast, in the present study, the bicarbonate ion concentration as well as C_T_ concentration are not correlated with any of the physiological processes measured (data not shown). Moreover, the present study shows that pH is not the main driver of the observed decreases in developmental success and growth rates. For instance, a decrease of pH_T_ to ∼7.6 (T5; below the projected levels for the end of the present century) had no significant effect on these larvae with similar developmental success and growth rates as compared to control conditions. It must be stressed that, for this treatment, *A*_T_ was artificially increased in order to maintain a seawater saturation state with respect to aragonite above 1. This saturation state depends on the availability of both Ca^2+^ and CO_3_^2−^ ions. Increasing Ca^2+^ concentrations in order to artificially maintain Ω_a_ above 1 (T4) had no beneficial effect on the larval development of oysters. As Ca^2+^ concentrations are not limiting in seawater (∼10 mmol kg^−1^), the main factor governing the growth of oyster larvae in our study was CO_3_^2−^ ion concentration. Several studies have already shown limited effects of calcium addition above 10 mmol kg^−1^ with coral calcification rates reaching a plateau at these concentrations [24], [25]. The relationship between measured parameters (developmental success, shell length and area and incorporated calcium) and the availability of CO_3_^2−^ showed that decreasing CO_3_^2−^ levels only had significant effects on the larval development below CO_3_^2−^ levels corresponding to aragonite saturation. On one hand, this is in contrast with results from Gazeau et al. [11] that showed that D-veliger shells of the blue mussel (*Mytilus edulis*) were 5±1% smaller following a 0.25–0.34 pH unit decrease corresponding to supersaturated conditions with respect to aragonite. On the other hand, Gazeau et al. [11] showed that developmental rates into viable D-shaped larvae at this pH level were not significantly altered, a finding which is consistent with present results. Accordingly, oyster larvae appear more resistant than blue mussel larvae to a decrease of pH as long as CO_3_^2−^ concentrations remain above the aragonite saturation level. Further decreasing CO_3_^2−^ concentrations below CO_3_^2−^ values corresponding to aragonite saturation has dramatic consequences as only ∼60% and ∼20% of the embryos had developed to viable larvae at CO_3_^2−^ concentrations of 50.4±3.3 and 40.0±2.4 µmol kg^−1^, respectively, as compared to more than 90% in the treatments exposed to CO_3_^2−^ supersaturated conditions. In the field, the different pressures exerted by the environment and predators result in considerable mortality rates, during the free-swimming larval period, possibly approaching 99% [26]. An additional decrease in developmental success as observed in the present study, under CO_3_^2−^ concentrations below aragonite saturated conditions, could therefore compromise the survival of the populations.

Most calcifying species, including mollusks, are able to concentrate Ca^2+^ and CO_3_^2−^ ions at the site of calcification [27] and should therefore be able to regulate calcification rates under suboptimal concentrations of Ca^2+^ and CO_3_^2−^. The fact that, even under CO_3_^2−^concentrations below aragonite saturated conditions (T3 and T4, assuming calcium addition has no effect in T4), some larvae were able to produce a shell highlights the efficiency of regulatory mechanisms. However, the percentage of embryos developing to viable D-veliger larvae and the shell sizes of these viable D-veliger larvae were smaller, suggesting that the regulation is not efficient enough to compensate for the low CO_3_^2−^ ion availability below aragonite saturated conditions. Nevertheless, many molluskan species are adapted to and able to survive under low alkalinity conditions such as those in freshwater ecosystems. In the marine environment, bivalve growth has been reported by Tunnicliffe et al. [17] under extremely undersaturated conditions prevailing close to deep hydrothermal sites, although shell growth rates were significantly lower than in non-acidified areas. Recently, Thomsen et al. [28] have shown that blue mussels are actively growing in a bay of the Western Baltic Sea naturally enriched with high CO_2_ water, and also juvenile recruitment occurs in summer time coinciding with low pH levels and aragonite undersaturated conditions. In the Oosterschelde tidal inlet (1998–2006, monthly measurements, 5 stations), surface pH_NBS_ varied annually between 8.00 and 8.24, while *A*_T_ varied between 2334 and 2567 µmol kg^−1^ (data not shown). The organisms inhabiting this ecosystem are therefore never exposed to corrosive waters and are even used to relatively high calcium carbonate saturation levels, especially in spring at the time of recruitment. Whether the organisms inhabiting environments with relatively high calcium carbonate saturation levels will be able to adapt to the anticipated decreases in pH and saturation levels in the coming decades remains an open question. According to the present results, the effects of ocean acidification on larvae of *Crassostrea gigas* from the Oosterschelde estuary during the first 3 days of development are not significant as long as CO_3_^2−^ concentrations remains above aragonite saturated conditions. Due to relatively high levels of total alkalinity in this area, it is not expected that seawater will become corrosive for aragonite following a decrease of 0.3 to 0.4 pH unit. However, the present study only focused on the developmental period between embryos and D-veliger larvae, there is still a need to perform experiments on the full larval development of this species and to investigate the response of other crucial physiological processes that have not been considered in the present study such as respiration and excretion.
